# The arena or E-games triathlon as a unique real world and virtual mixed-model endurance sports event

**DOI:** 10.3389/fspor.2024.1444385

**Published:** 2024-07-18

**Authors:** Paul J. Stapley, Romuald Lepers, Tim Heming, Vincent Gremeaux

**Affiliations:** ^1^ Faculty of Science, Medicine and Health, School of Medical, Indigenous and Health Sciences, University of Wollongong, Wollongong, NSW, Australia; ^2^ Faculté des Sciences du Sport, Inserm CAPS U1093, Université de Bourgogne, Dijon Cedex, France; ^3^ Heming Media Ltd., Cheltenham, United Kingdom; ^4^ CHUV—Département de l’Appareil Locomoteur (DAL), Médecine du Sport et de L'exercice et Médecine Physique, Swiss Olympic Medical Center, Lausanne, Switzerland; ^5^ Institute of Sport Sciences (ISSUL), University of Lausanne, Lausanne, Switzerland

**Keywords:** triathlon, Esports, physiology, media, Olympics

## Abstract

The sport of triathlon has evolved to become a discipline comprising races of different lengths and formats. It has also undergone significant growth in popularity and exposure with several variations in format from the classic swim – bike – run combination attracting significant television and media coverage. Since 2021 an original and unique format called the Arena Games Triathlon (or in 2024 the E-Games) has evolved that involves athletes competing against each other in swimming, cycling and running combining both the real and virtual worlds in one race. This model of endurance sport is currently unique, provides instantaneous data on performance and has the potential to be a tool for performance prediction, talent selection and sport development. The goal of this perspective paper is to provide context around the emergence of the Arena Games Triathlon series, describe the format of this type of racing, outline how it has the potential to drive training and evaluation of triathletes and discuss the attractiveness of its future inclusion as an Olympic discipline.

## 1 Introduction

As a multisport discipline, triathlon has traditionally been a swim-bike-run combination of varying distances from super sprint, sprint, standard (often known as Olympic) distance to half Ironman and Ironman, as well as all-terrain events consisting of a swim, mountain bike cycle leg and ending with a trail run (1). Participation in this type of multisport has developed considerably among all age groups (2). Triathlon was incorporated into the Olympics in 2000 in Sydney with a standard distance 1.5 km swim, a 40 km bike ride and a 10 km run event for both men and women. It has featured in every Olympic Games since, expanding to include a four-person team relay event in Tokyo. Olympic inclusion has helped increase the recognition of triathlon as a global endurance sport.

Before the Sydney Olympics however, the sport had experienced a brief period of expanded media coverage and significant sponsorship through major events that incorporated non-standard formats. During the 1990s for example, triathlon in Australia embraced a different format consisting of shorter distances and changing the order of the three disciplines between heats. The Formula 1 St George Sprint Series was in certain respects ahead of its time as it featured on prime-time TV in Australia and attracted major media interest (3). The F1 series eventually disappeared but other events such as the Super Sprint Tri Series, a 300 yd swim, 10 mi bike leg and 1.5 mi run all executed twice, occurred in the US between 2010 and 2013 (3). In 2017, the non-traditional format of triathlon racing re-emerged through the staging of a unique three event race called Super League Triathlon which from 2024 was rebranded as Supertri. The first of these events was held on Hamilton Island, Australia. This format of racing led to an annual yearly series from 2019 in venues around the world, such as Malta, Jersey and London (UK), Malibu (USA), Munich (Germany), Toulouse (France), and Neom (Saudi Arabia).

During the COVID-19 pandemic period competitive outdoor triathlon was either cancelled or postponed. Subsequently, the organizers of Supertri series innovated with a COVID-secure solution with athletes racing in Olympic-sized swimming pools and cycling on bike trainers and treadmills connected to a virtual racing platform called Zwift (4). The racing was named the Arena Games Triathlon (AGT) and combined real swimming with mixed reality cycling and running and since 2021 has evolved into a new form of triathlon competition. The primary aim of this article is to give our perspective that this new Esports format of triathlon boosts interest in the sport and could provide an evaluative model of performance. We do so by defining the format and mixed-reality technology adopted by Supertri when hosting AGT, document how it has generated significant interest via media exposure, define methodological considerations around AGT and suggest it could be used as a unique model for talent identification and the monitoring of training effects. We also suggest that this model of triathlon may be adopted as a future Olympic Esports discipline.

## 2 Arena games triathlon—a unique Esports hybrid endurance sport

The emergence of AGT as an event could only have occurred due to the existence of a software platform enabling mixed-reality (actual running or cycling within a “virtual world”). In fact, in 2021, partially driven by the COVID-19 pandemic, mixed-reality cycling had reached maximum levels of participation (5) using the platform called Zwift (6). This mode of cycling involves the participant acting within their avatar which is driven by the physical power generated at the pedals measured by a power meter incorporated into a trainer upon which an athlete's own bike is mounted. Power is measured in watts and the speed of the avatar is the result of a calculation using the athlete's body mass and height (6). As multiple users can unite on the same cycle route at the same time, the system accounts for “drafting” behind another cyclist (slipstream effect), different course profiles (hills, corners, etc.) and can reproduce real cycle routes. The Zwift system uses an algorithm to define how the athlete's performance is translated into a virtual world. The platform became so popular that in December 2020 the governing body the Union Cycliste Internationale (UCI) hosted the first Esports world cycling championship (7).

In 2022 and 2023, the AGT series comprised 3 events between the cities of London, Singapore, Munich, Montreal and Sursee. Thirty male and female athletes raced in qualifying heats of two stages of a 200 m swim, 3.8 km virtual cycle and 1 km virtual run. The winners and runners-up of the heats joined either the fastest qualifiers or winners of a repechage event in a final featuring 10 athletes and composed of 3 stages: stage 1 (swim – bike – run), stage 2 (run – bike – swim) and stage 3 (swim – bike – run). For stage 3, athletes started at intervals based on their cumulative times from stages 1 and 2. In 2024, this format was used for the AGT or E-Games World Championship in London. For the finals, total finish times were between 34 and 35 min for the men (11–12 min per stage) and 37–38 min for females (12–13 min per stage).

The running leg of the AGT is also conducted in the virtual Zwift world. However, the use of regular motorised treadmills would mean that any changes in running speed required during races can only be made by changing the speed of the motor that drives the running belt. This would not replicate a “live” race scenario where the athlete's foot speed dictates the pace at which they are moving. The AGT series therefore adopted the use of non-motorised treadmills (NMT) because they are participant-driven and allow runners to self-regulate their pace (8).

## 3 The media impact of arena games triathlon

The model of Supertri introduced by Michael Dhulst, Leonid Boguslavsky and four-time triathlon world champion Chris McCormack has brought a shorter and more dynamic form of triathlon to followers of the sport. Races are well designed to suit TV broadcast, often feature spectacular locations and provide unique athlete perspectives (interviews) prior to, during and after races. In 2022, Supertri's four events attracted 51 million viewers across more than 20 broadcast partners in 200 markets and data from 2023 suggest an increase by 25% of viewership in Europe and considerable growth in the Middle East with Supertri adding Arabic language commentary to events (9). In 2022, AGT itself attracted 21.7 million viewers globally and developed €94 million ($US 97 million) worth of media value. The race series was shown in 136 countries with more than 9,000 broadcast hours, half of which were in Europe and North America (10). In 2023, AGT saw a 4% increase in its live audience to 4 million and total broadcast reach rose from 21.7 million in 2022 to 25.4 million. Moreover, 95% of stadium tickets at the three AGT events (Montreal, Sursee and London) were sold (11).

How does the media exposure of AGT compare to other traditional formats of triathlon? A comparison may be made with the World Triathlon (WT) series races that feature short Sprint, Standard (Olympic), 2-day Eliminator races (e.g., Tiszaujvaros, Hungary) and Mixed Team Relay events. In 2022 and 2023 respectively there were 17 and 23 WT organised events (either World Triathlon Championship Series or World Cup races) compared to three AGT races in both 2022 and 2023. World Triathlon attracts approximately 360,000 viewers annually on their subscription-based TV platform and 750,000 followers across their Instagram, Facebook and Twitter accounts (12). In comparison, Supertri (comprising AGT) in addition to the viewership reported above attracts over 1,235,000 followers on social media. These statistics would suggest that Supertri (including the ESport of AGT) is clearly the most viewed format in short course triathlon regardless of the lower number of annual events compared to WT.

## 4 Logistical and methodological considerations of using virtual platforms for cycling and running in arena games triathlon

Several significant logistical challenges have been overcome to host a now successful AGT race series. These challenges include (but are not limited to) pool timing, transition to the Zwift platform for cycling and running and above all, making it into a coherent, viewer-friendly interface while minimising errors, all within a very humid atmosphere. The attractiveness of the AGT is that it is a format that is not influenced by weather, water quality, or road closure approvals as other conventional (outdoor) triathlons are.

As the swim leg in AGT is performed in the real world and swim times can be quantified as they are for regular swim meets (starting procedures, timing touchpads, etc.) it is not subject to known inaccuracies of both virtual cycling and running. Whiting et al. (13) have previously documented that questionable accuracy is one of the most obvious weaknesses of the cycling platform, Zwift. For the cycling leg in AGT to be virtual, athletes race each other using their own bicycles mounted on TacX cycle ergometer (Garmin LTD, Olathe, KS, USA) trainers. This set-up has been reported to result in a +/−1%–3% variance in accuracy of power output (14), therefore if the variance is not consistent between athletes it can result in small differences that can potentially determine results at the elite level. Interestingly however, Westmattelmann et al. (15, 16) showed through comparisons of real vs. virtual cycling races (e.g., Virtual Tour de France vs. German national and Pro Tour races) that Zwift data is reliable both for male and females and performances in real and mixed-reality virtual worlds match. Bjärehed and Bjärehed (17) also showed that the power outputs measured by the Zwift virtual platform derived from the trainers agreed with crank mounted power meters. Therefore, despite the variance, the Zwift and indoor trainer set-up allows for competitive usage.

Another issue and risk of the virtual Zwift/TacX platform is the possibility of “dropouts” or even system failure in data streaming (14). Bluetooth or ANT+ connectivity is used between devices and in large arenas such as Olympic swimming pools, signal drop-out is a distinct threat to the integrity of the data used to determine positioning when cycling and running. Indeed, running on the NMT in AGT has seen several high-profile drop-outs resulting in inaccurate avatar run speeds (e.g., Gordon Benson, Munich AGT 2022 and Aurelien Raphael, London AGT 2022).

A significant issue, already identified in cycle Zwift racing (18) is that of cheating using so-called “cyber, or digital-doping”. For estimated, actual power output and speed generated on Zwift, body mass and height must be manually entered into the platform to calculate watts per kilo and a drag coefficient, respectively. Alongside the power generated through the pedals, these calculations are the main determinant of avatar speed, and their falsification can change the outcome of the cycle leg. Arena Games Triathlon adopts a “weigh-in” session, usually the day before the race which leaves open (as in boxing for example), the option for athletes to shed weight, sometimes by spending time in a sauna before the weigh-in, meaning that power-to-weight ratios might not be a true reflection of race conditions on the day. Zwift itself has adopted measures to counter cyber-doping in mixed-reality cycling by creating the Zwift Anti-Doping Agency (ZADA) and Richardson et al. (19) has detailed several recommendations to counter digital doping. These include: (1) Implementing Zwift-specific anti-doping rule violations (Digital Doping Rule Violations, or DDRVs) to tackle data manipulation and software hacking, (2) Adding the DDRV to update and improve the current 3-tier ban system, (3) Seeking approval for the UCI to hear digital-doping cases by the Court of Arbitration for Sport, the expert forum on doping matters, and (4) the option to dual-recording from cycle mounted power meters with trainer-recorded power to identify any incorrect calibration values and minimize the manipulation of power data. The above issues and solutions are applicable to the cycle leg of AGT and would greatly benefit its integrity.

Other considerations of using NMT in virtual racing are the physiological and perceptual demands associated with them compared to simulated real world running. Schoenmakers and Reed (8) compared NMT running with running on a MT at 1% gradient (deemed equivalent to matching the VO_2_ demands of running outside) across a range of running speeds. Their study showed that NMT elicits higher relative oxygen uptake and ratings of perceived exertion at all speeds compared to MT running. Therefore, there should be some consideration in AGT of the higher efforts required to run on a NMT, compared to racing in the real world. This is particularly important to consider in AGT as the elevated ambient temperature of the pool setting can lead to dehydration and additional physiological strain as airflow around the athlete is reduced because they are comparatively static during both cycle and run legs.

## 5 Arena games triathlon: a unique training and evaluation model of triathlon?

Talent selection in triathlon among large federations is presently structured around outdoor sprint or super sprint length series races or tests of timed pool swims and track runs. For example, since 2008, the French Triathlon Federation have maintained a classification of 12–13- and 14–15-year-olds using cumulative times over a 200 m swim and 1,000 m run, 16–17 year olds over a 400 m swim and 1,500 m run and 18–20 year olds (juniors) over a 400 m swim and 3,000 m run (20). Times are compared with standards for selection to integrate development centres. However, it is unclear if these tests are predictive of future success in elite-level triathlon. One study by Cuba-Dorado et al. (21) found that results from talent identification tests conducted by the Spanish federation (FETri) were low in predicting subsequent performance in 247 athletes. Therefore, a possible alternative for talent identification using the mixed reality AGT model could be adopted by trainers and federations to simulate the requirements of swim, bike and run and provide an overall composite score across the three disciplines. Coaches and selectors would receive a wealth of data (heart rate, power output, cadence), upon which estimations of aerobic capacity and suitability for triathlon could be determined. The AGT model provides opportunities for field-based studies using in-field technology, for example VO_2_ Master (Vernon, BC, Canada) or core body temperature measures. Longitudinal studies could easily be conducted using the model of AGT year-on-year to plot progression and enable coaches to highlight athlete strengths and weaknesses and tailor training programs. The attractiveness of AGT is that it requires high intensity bouts separated by short recovery periods, which may also provide a solid base for future short-course triathlon performance.

## 6 Arena (or E-) games triathlon: integration with existing competitive structures and a future potential Olympic discipline?

The AGT has gained significant recognition within existing competitive frameworks since its inception. The WT annual race series awards points to athletes for AGT series events that can amass towards their overall WT ranking. There has been successful cross over of athletes from other formats to AGT (e.g., Lionel Sanders in 2023) generating significant media exposure for the sport. Supertri have also scheduled AGT events to dovetail with the WT short course season by holding events in late winter or spring which maximises the chances of attracting high calibre athletes. Overall, AGT seems to provide little threat to existing competitive structures.

Given its distinctiveness as a mixed real/virtual sport, the AGT model would represent a unique potential Olympic discipline. Discussions and proposals for the creation of an Esports Olympic Games have developed considerably over the past few years. In March, 2023 a global virtual and simulated sports competition created by the International Olympic Committee (IOC) culminated with the Olympic Esports series in Singapore (22) with duathlon (run – bike – run) being included (23). Indeed, the President of WT, Marisol Casado hinted that the mixed-reality format may expand saying:

“*Being able to feature as an Exhibition Event at this year's Olympic Esports Week is a historic moment for our sport and one that builds on our hard work and collaboration with Super League in creating a World Championships for the Arena Games competitions over the past two seasons of our partnership…we can't wait to shape the future of the format further.*” (23)

Moreover, in October 2023, the IOC announced plans for the creation of an Esports Olympic Games (24), and in April 2024, Supertri held the first E-Games World Championships in London. Thus, AGT is already integrated into existing competitive frameworks with the support of the triathlon governing body (WT) to become an Olympic Esport.

## 7 Summary and future directions

This perspective paper has explained the history and evolution of non-standard triathlon events and the creation of a mixed-reality event called the AGT. The model is unique as it provides the viewer/spectator who can be both online or in the stadium, with an experience of elite sporting performance that spans the real and virtual worlds simultaneously with constant streaming of variables (heart rate, running speed, power output) that enhance the viewing experience. In addition to the exciting spectator appeal, the AGT model provides athletes, coaches, talent selectors and sports scientists with a unique perspective on the physiological demands of the sport of triathlon. Coupled with other measurements of physiological variables the model of AGT can evolve to enhance our knowledge of the physiological requirements of each of the three sports and most importantly, transitioning between swimming, cycling and running which are key determinants of success in short course triathlon. Finally, given its unique real and virtual modes, the AGT would provide an exciting Esports Olympic discipline. Future research should focus on the use of AGT performance to predict subsequent success in triathlon, the physiological demands placed on athletes during AGT races through in-field testing and the influence of this mixed-reality model upon the popularity of the sport.

## Data Availability

The original contributions presented in the study are included in the article/Supplementary Material, further inquiries can be directed to the corresponding author.
